# Anal fistulotomy with one-stage shaped skin grafting for intersphincter anal fistulas: study protocol on a multicentre randomised controlled trial

**DOI:** 10.1186/s13063-023-07495-7

**Published:** 2023-07-22

**Authors:** Yongchang Zhao, Wei Xie, Xiaoshuo Wu, Xiaolan Li, Jinyan Guo, Qiurui Cao, Jiadi Liang, Xin-lin Chen, Wentao Zhao, Feng Sun, Hongjie Li, Weimin Luo, Yuying Li

**Affiliations:** 1 Department of Anorectal Surgery, the First Affiliated Hospital of Guangzhou University of Chinese Medicine, Guangzhou, 510000 China; 2 Department of Gastrointestinal Surgery, Guangzhou Panyu Hexian Memorial Hospital, Guangzhou, 510000 China; 3 Department of Anorectal Surgery, Dongguan People’s Hospital, Dongguan, 523000 China; 4 Department of Anorectal Surgery, Jiangmen Wuyi Hospital of Chinese Medicine, Jiangmen, 529000 China; 5 School of Basic Medical Science, Guangzhou University of Chinese Medicine, Guangzhou, 510000 China

**Keywords:** Anal fistula, Anal fistulotomy, Randomised controlled trial, Skin graft

## Abstract

**Background:**

Anal fistulas are mainly treated via surgery. They can be difficult to treat without surgical intervention. Numerous procedures, such as fistulectomy and fistulotomy, are performed to treat anal fistulas and achieve good effects. However, the wounds created through fistulectomy and fistulotomy take a long time to heal. Therefore, a multicentre randomised controlled trial (RCT) is proposed to study the efficacy of one-stage shaped skin grafting at the surgical wound to heal low simple intersphincter anal fistulas.

**Methods:**

This study is a multicentre, hospital-based RCT. It will be performed at three hospitals. A total of 104 patients with low simple intersphincter anal fistulas who meet the inclusion criteria will be included in this trial and will be allocated randomly to two groups (test and control groups). The patients in the test group will receive one-stage anal fistulotomy surgery combined with shaped skin grafting, and those in the control group will undergo anal fistulotomy only. All the operations will be performed by attending colorectal surgeons or surgeons of a higher level. Effectiveness and safety indicators will be observed, recorded and analysed.

**Discussion:**

Anal fistulotomy can heal low simple intersphincter anal fistulas effectively and safely with a low recurrence rate. Skin grafts promote wound epithelisation significantly. We believe that skin grafting can treat low simple intersphincter fistulas with a short healing time.

**Trial registration:**

Chinese Clinical Trial Register, ChiCTR2000039174. Registered on 28 October 2020.

## Administrative information

**Table:** 

Title {1}	Anal fistulotomy with one-stage shaped skin grafting for intersphincter anal fistula: study protocol for a multicentre random controlled trial
Trial registration {2a and 2b}	2a: The technique of anal fistulotomy with shaped skin harvesting and grafting at the surgical wound in one stage for healing the low simple intersphincter anal fistula: study protocol for a multicentre random controlled trial 2b: Chinese Clinical Trial Register, ID: ChiCTR2000039174. Registered on 28 October 2020
Protocol version {3}	15 April 2020, Version 5.0
Funding {4}	This study was funded by the Innovation Strong Hospital Project of the First Affiliated Hospital of Guangzhou University of Chinese Medicine and Science and Technology Projects in Guangzhou
Author details {5a}	^a^Department of Anorectal Surgery of the First Affiliated Hospital of Guangzhou University of Chinese Medicine, Guangzhou 510,000, China;Doctorate with Equivalent Academic Qualification of Guangzhou University of Chinese Medicine ^b^Doctorate with Equivalent Academic Qualification of Guangzhou University of Chinese Medicine;Department of Gastrointestinal Surgery, Guangzhou Panyu Hexian Momerial Hospital,Guangzhou 510,000, China ^c^ Department of Anorectal Surgery of Dongguan People’s Hospital,Dongguan 523,000, China ^d^Department of Anorectal Surgery of Jiangmen Wuyi Hospital of Chinese Medicine,Jiangmen 529,000, China ^e^School of Basic Medical Science, Guangzhou University of Chinese Medicine,Guangzhou 510,000, China ^f^Department of Anorectal Surgery of the First Affiliated Hospital of Guangzhou University of Chinese Medicine, Guangzhou 510,000, China
Name and contact information for the trial sponsor {5b}	The First Affiliated Hospital of Guangzhou University of Chinese Medicine, 16^th^ Jichang Road, Guangzhou 510,405, China Guangzhou Municipal Science and Technology Bureau,1^st^ Fuqian Road, Guangzhou 510,030, China
Role of sponsor {5c}	The sponsors played no part in study design, collection, management, analysis and data interpretation; report writing and deciding to submit the report for publication

## Introduction

### Background and rationale

Anal fistulas, which are common in colorectal diseases, affect approximately 1/10,000 of the normal population every year, according to Corson’s report [1]. A fistula is a pathological connection between two epithelialised openings. An anal fistula is a granulation tract between the anal canal or rectum and the skin around the anal verge. Approximately 80% of anal fistulas develop secondary to cryptogenic abscesses arising from infected anal glands, which can spread to other parts in many directions and represent chronic perianal sepsis conditions [1].

A series of surgical or nonsurgical therapeutic options for anal fistulas have been developed. Nonsurgical methods include antibiotics, anal fistula plugs and fibrin glue. Numerous surgical procedures are available and include two types: impairing or reserving the anal sphincter, such as laying the anal sphincter open, fistulotomy, fistulectomy, loosening or cutting the seton, ligating the intersphincteric fistula-tract, video-assisted anal fistula treatment and fistula-tract laser closure. However, the ideal surgical technique for anal fistula treatment should aim to deracinate the internal opening, which often leads to sepsis, and the granulation tract then promote postoperative wound healing while preserving sphincter function and anal continence because maintaining normal continence relies on the complex interaction of the anal sphincter and pelvic floor innervations [2]. Procedures for impairing and preserving anal sphincters in anal fistulas have postoperative recurrence rates of 0–65% and incontinence rates of 0–63%. In radical techniques that involve cutting additional anal sphincter muscles, the recurrence rate decreases, and the incontinence rate increases accordingly [3]. Laying simple and low anal fistulas open directly is relatively effective and safe and is widely accepted by surgeons [4]. Anal fistulotomy, which involves the division of pathogenic tracts, has been applied to treat subcutaneous and intersphincteric anal fistulas and has an excellent healing rate of over 90% [3, 5]. However, the risk of faecal incontinence increases with the extent of the division of the external anal sphincter [6–8].

In addition to concerns about anal sphincter damage and even faecal incontinence, long postoperative wound healing times are another major challenge posed by anal fistula surgeries. Most techniques have wound healing times of approximately 4 weeks, and some even have wound healing times of 8 weeks. In Ahmed’s study, the patients who received one-stage fistulectomy had average wound healing times of approximately 3–4 weeks [9]. The continuous purulent discharge of the wound and other complications during wound healing may make the patient uncomfortable, affect their quality of life considerably and even delay their return to work.

Anal fistulotomy is an effective and safe technique for the treatment of simple and low anal fistulas. It has a healing rate of over 90%. However, its postoperative recovery time, especially its wound healing time, is very long. We have made some attempts to shorten the wound healing time of this technique. Our early observations showed that skin grafting at the wound area after fistulotomy promotes wound healing significantly. We grafted full-thickness skin from the area of the anal fistula and then performed a fistulotomy in one stage. We anticipate that this technique will shorten wound healing time because numerous studies have demonstrated that skin grafting is an effective treatment for the rapid coverage of ulcers [10]. A multicentre randomised controlled trial (RCT) is proposed to study the efficacy of the method of one-stage anal fistulotomy with shaped skin graft (SSG) at the surgical wound to heal low simple intersphincter anal fistulas and confirm our supposition about this method.

### Objectives

This trial aims to confirm the efficacy of one-stage SSG at the surgical wound after anal fistulotomy for low simple intersphincter anal fistulas.

### Trial design

This randomised, double-blinded, parallel, superiority trial will be completed at three research centres, including the First Affiliated Hospital of Guangzhou University of Chinese Medicine (GZUCM), Wuyi Hospital of Traditional Chinese Medicine and Dongguan People’s Hospital, over 2 years. A total of 104 patients with low simple intersphincter anal fistulas who meet the study criteria will be randomised (1:1) to the test and control groups. The intervention will last for the whole duration of the hospital stay with a follow-up of 6 months. Informed written consent will be obtained prior to the commencement of this study. All enrolled subjects will be fully informed about the purpose, process and possible risks of the study.

## Methods: participants, interventions, and outcomes

### Study setting

The study will be completed at three research centres.

### Eligibility criteria

**Fig. 1:**
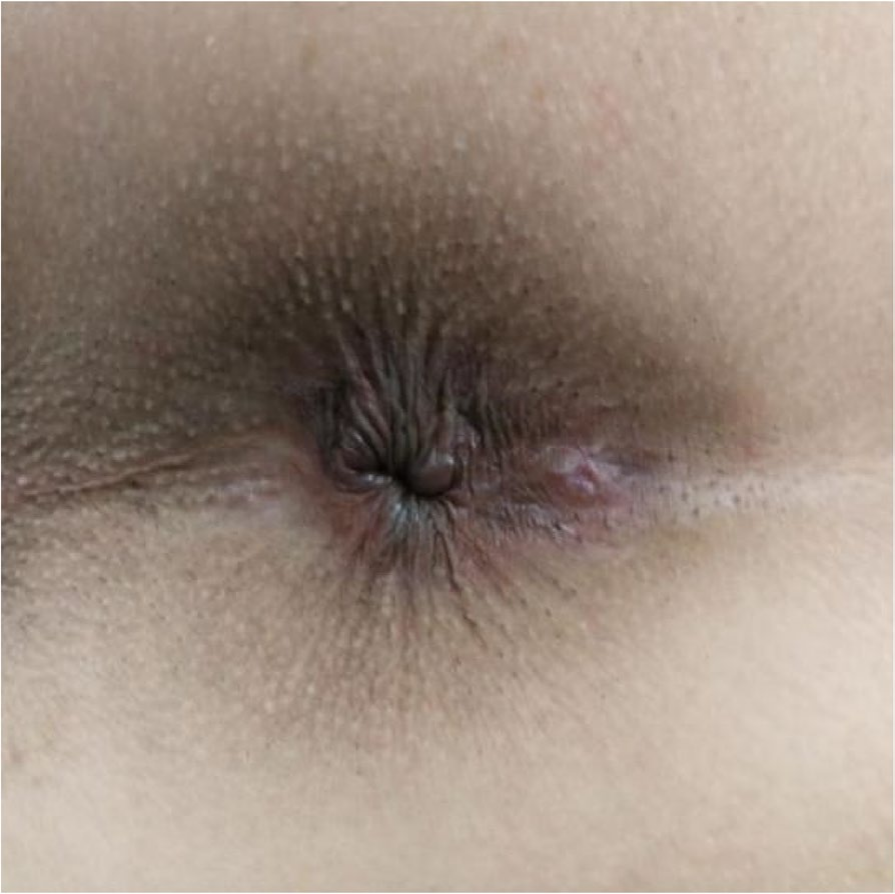
Low simple intersphincter anal fistula

Patients with low simple intersphincter anal fistula (Fig. 1) will be enrolled from the First Affiliated Hospital of GZUCM, Wuyi Hospital of Traditional Chinese Medicine and Dongguan People’s Hospital.

#### Inclusion criteria

1. Patients who received rectal–anal magnetic resonance imaging (MRI) examination and were diagnosed with low simple intersphincter anal fistula in accordance with ‘Chinese Colorectal and Anal Surgery’ compiled by Wang Jianping and published by People’s Medical Publishing House in 2014.
2. Patients aged between 18 and 60 years.
3. Patients with normal anal morphology and function and without anal abnormality and incontinence.
4. Patients with internal and external openings and tract distribution between 5 and 7 o’clock around the anal verge in the lithotomy position.
5. Patient with body mass index (BMI) < 30.
6. Patients who agreed to participate voluntarily in this study, signed an informed consent form, and had good compliance. For example, patients who can understand the study, agreed to accept and complete all the interventions of the study, and can be followed up in the stipulated timeframe.

#### Exclusion criteria

1. Patients with anal trauma, inflammatory bowel disease, malignant tumours, HIV, tuberculosis, actinomycotic infection and other perineal complications.
2. Special patients who are pregnant and lactating or patients with respiratory, digestive, circulation, blood system disorders, liver and kidney dysfunction or other disorders.
3. Patients with diabetes and/or other special factors (radiation damage and burns) that affect wound healing.
4. Patients who are under acute perianal sepsis conditions.
5. Patients with abnormal anal morphology and function.
6. Patients whose internal opening and tract are above the dental line by over 1 cm.
7. Patients with more than one internal or external opening and tract.
8. Patients with contraindications to MRI.
9. Patients who cannot understand and disagree to join the study and cannot be followed up.

#### Withdrawal criteria

1. Patients who do not meet the inclusion criteria after inclusion in the study.
2. Patients who are not given the established treatment of the study.
3. Patients without personal information or with untruthful personal information.
4. Patients with serious adverse reactions during treatment, who voluntarily quit or who are found to be ineligible for the study by the investigators.
5. Patients without any treatment after inclusion.

### Who will collect informed consent?

Written informed consent from potential trial participants or authorised surrogates will be obtained by a colorectal surgeon at three research centres.

### Additional consent provisions for the collection and use of participant data and biological specimens

Not applicable.

## Interventions

### Explanation for the choice of comparators

Although anal fistulotomy is effective and safe, its postoperative recovery time, especially wound healing time, is very long. Our early observations showed that grafting at the wound shortened the wound healing time significantly compared with the technique without grafting. We proposed this study, which aims to compare anal fistulotomy with grafting with anal fistulotomy without grafting, to confirm our findings on grafting. A total of 104 patients with low simple intersphincter anal fistulas who meet the study criteria will be randomised (1:1) to the test and control groups.

### Intervention description

The patients in the test group will receive one-stage anal fistulotomy surgery combined with SSG, and those in the control group will undergo anal fistulotomy only. All the operations will be performed by attending colorectal surgeons or surgeons at a higher level. During this trial, all subjects will receive general treatment.

**Fig. 2:**
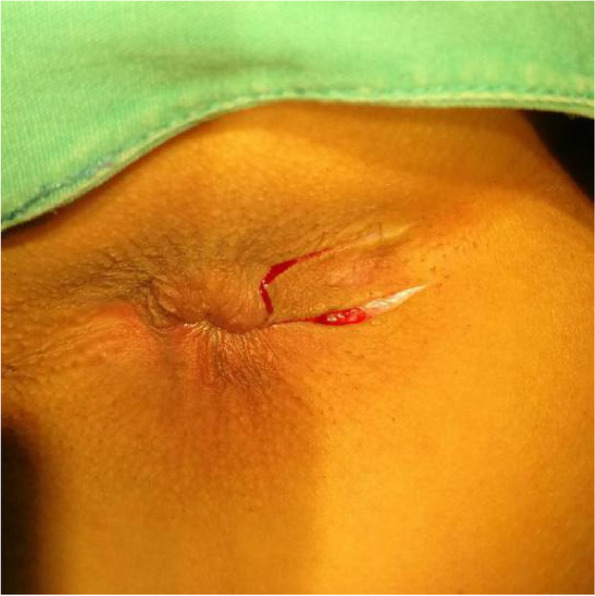
Incision and freeing of the skin

**Fig. 3:**
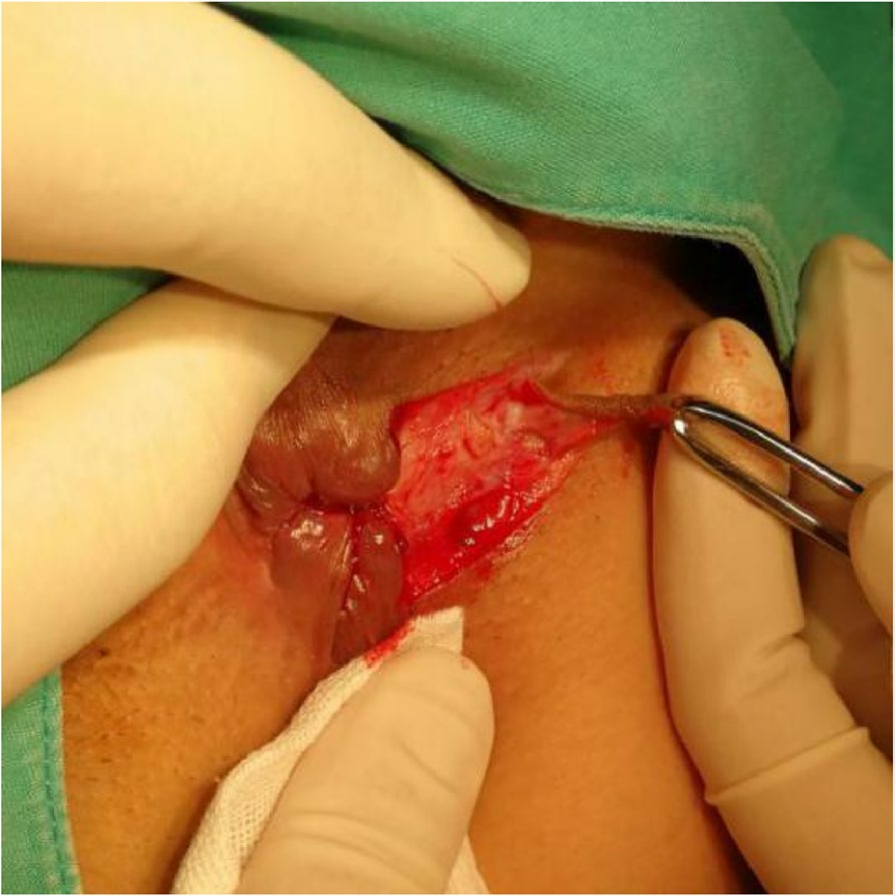
Incision and freeing of the skin

**Fig. 4:**
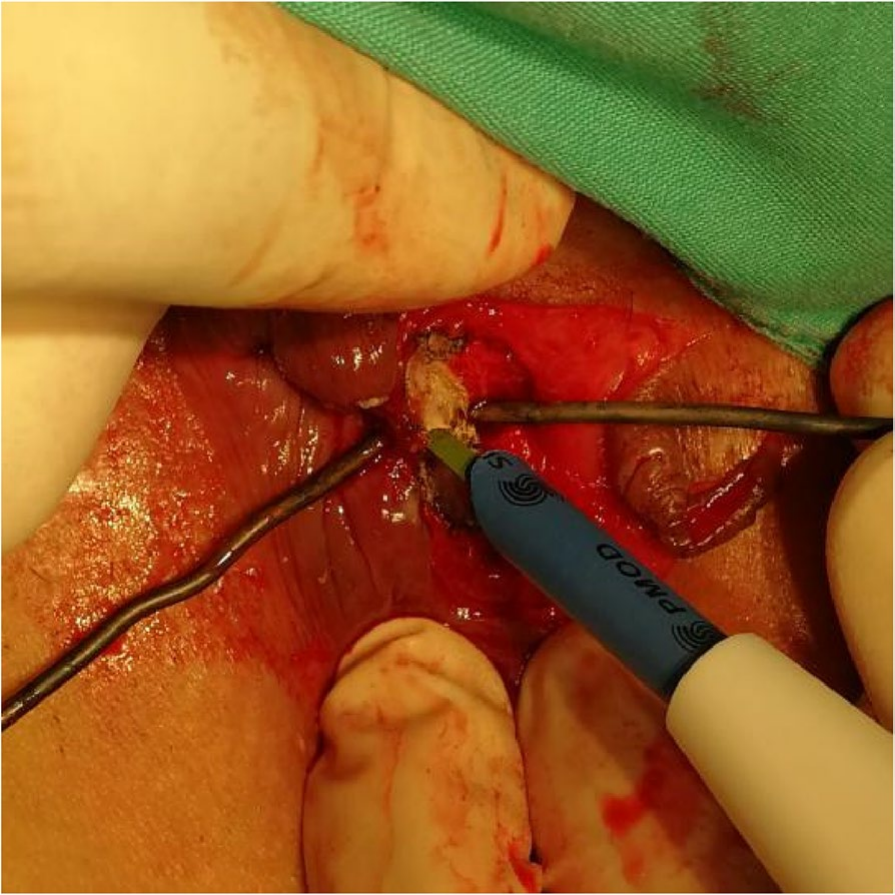
Opening of the tract

**Fig. 5:**
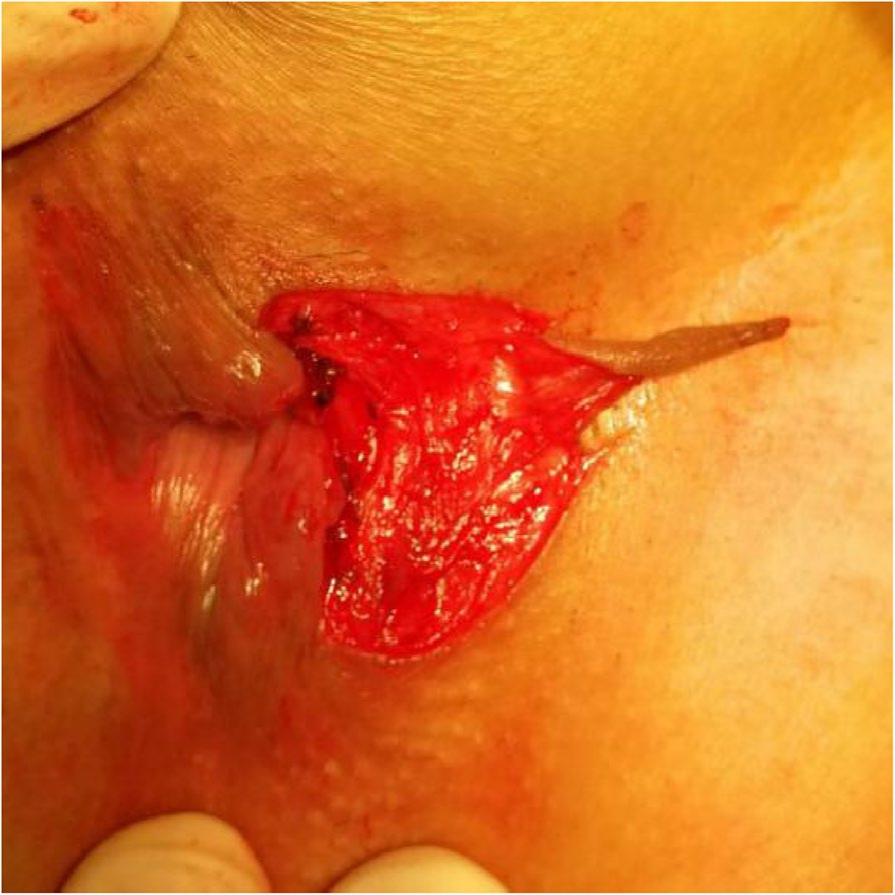
Scraping of the wound

**Fig. 6:**
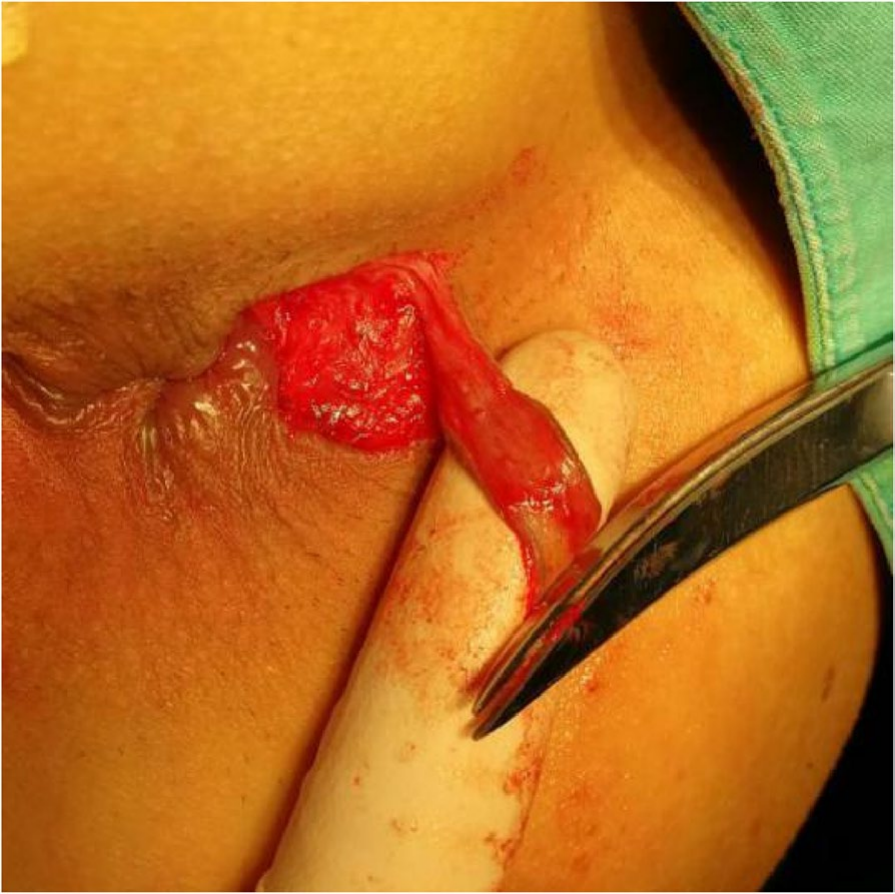
Obtaining full-thickness skin

**Fig. 7:**
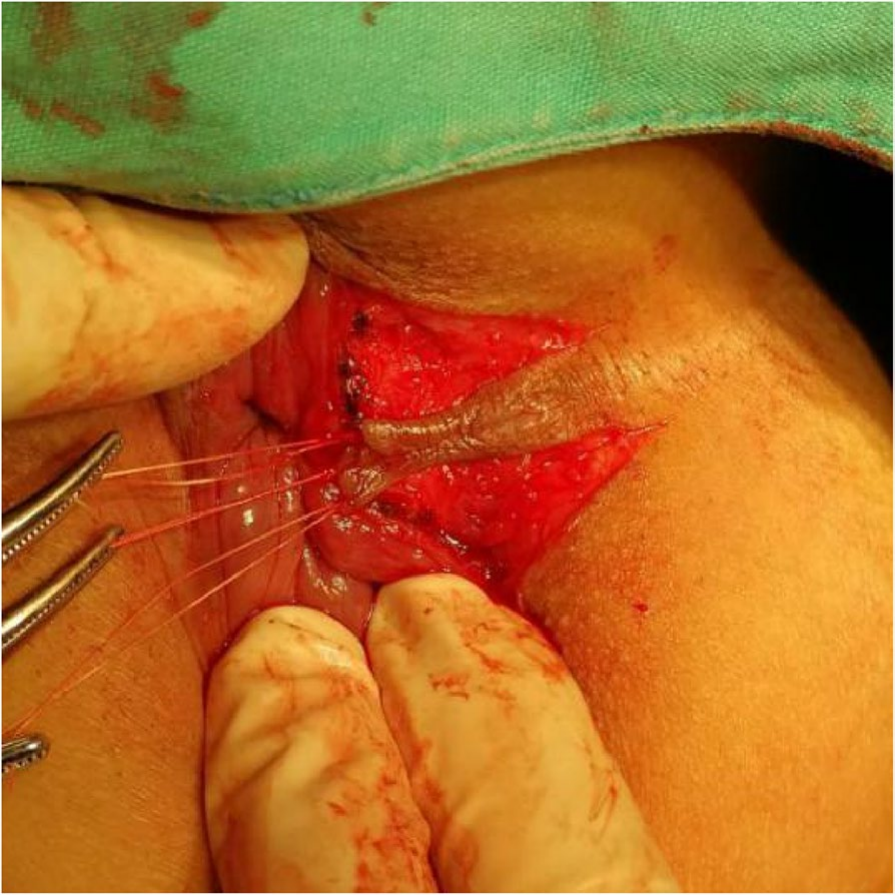
Grafting of the skin

**Fig. 8:**
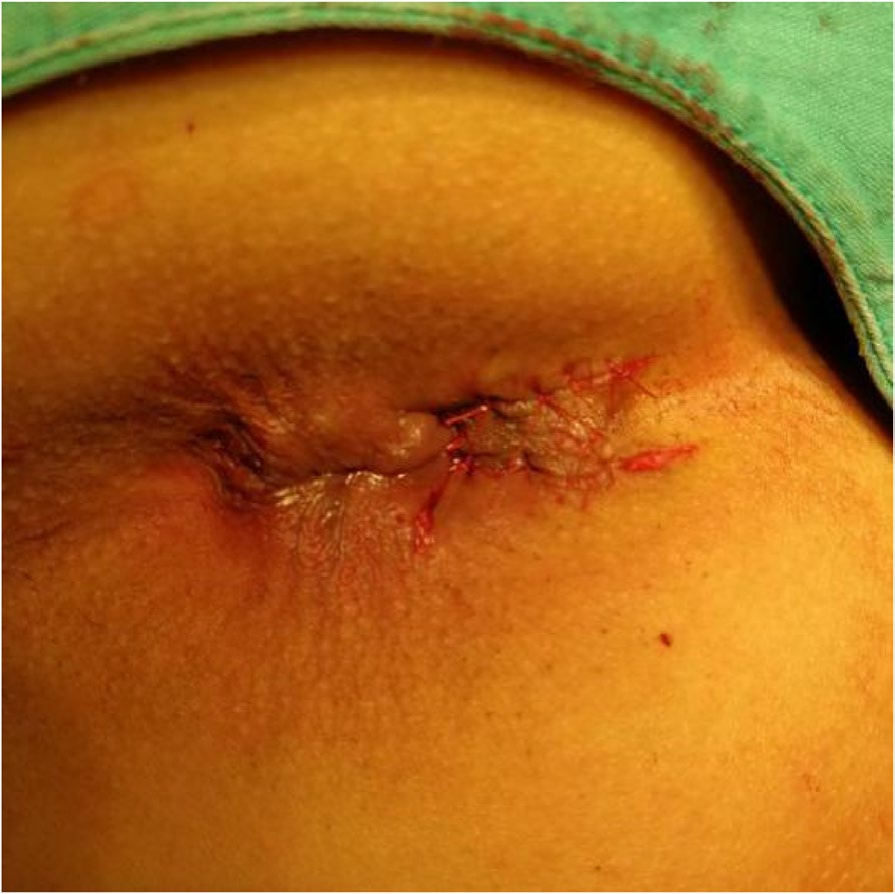
Completing the procedure

The patients in the test group will be on a semiliquid diet and take oral polyethylene glycol electrolyte powder for bowel preparation one night before operation. The patients will be placed in the lateral position after spinal anaesthesia, and their perianal skin will be sterilised. Their tract and internal opening will be probed by using a probe. Then, a strip of skin with a size of 1. 0 cm × 3.0 cm will be incised and freed below the dental line along the fistula and distal to the outer side of the anus without cutting its end (Figs. 2 and 3). The probed tract will be opened and scraped (Figs. 4 and 5), and the inflamed tissue will be removed. Some tissue will be retained for biopsy. Wound bleeding will be stopped by electrocoagulation if necessary, making the harvested skin from the surgical region into full-thickness skin (Fig. 6) and grafting it at the wound closely without tension by stitching with 4–0 Coated Vicryl (Figs. 7 and 8). The wound will be covered with petrolatum gauze and sterilised dressing. All the patients will be given celecoxib one piece at a time twice a day to relieve pain for 7 days. Patients will be advised to go on semiliquid diets and control bowel movements for 3 days. The wound will be cleaned gently with warm water after every bowel movement. Patients will be followed-up on the 3rd day, 7th day, 14th day, 21–22nd day, 27–29th day, 2nd month, 3rd month and 6th month postoperation in clinic visits.

The control group will be placed on the same diet and undergo the same preoperation bowel preparation as the test group. The patients in this group will receive a fistulotomy from eligible colorectal surgeons. Other postoperative treatments, such as changing wound dressing and follow-ups, are also the same.

### Criteria for discontinuing or modifying the allocated interventions

Rejection criteria: (1) Patients who do not meet the inclusion criteria after inclusion in the study. (2) Patients who are not given the established treatment of the study.

Shedding criteria: (1) Serious adverse reactions related to the study procedure. (2) Major mistakes in the clinical research protocol that make evaluating the efficacy of the technique difficult. Observation of a significant deviation in the implementation of a well-designed protocol.

Subject termination criteria: (1) Subject terminates spontaneously (e.g. withdrawal of informed consent). (2) Subject uses a combination of drugs or undergoes other operations within a nonprescribed range that may affect the judgement of efficacy and safety. (3) Indices of liver function: transaminase ≥ 3 times the upper limit of normality for > 1 week. Indices of renal function: Cr ≥ 1.2 times the upper limit of normality for > 1 week. (4) Subject develops adverse events or their condition worsens, leading the investigator to decide that the subject has to quit early. (5) Other investigators consider that the subject is unsuitable to continue and needs to quit. (6) Subjects who voluntarily quit or are found to be ineligible for the study by the investigators.

### Strategies to improve adherence to interventions

Subjects will be regularly called back to the hospital for follow-up. They will undergo laboratory tests, and pictures of their surgical wounds will be taken with their agreement.

### Relevant concomitant care permitted or prohibited during the trial

This trial will not impose special requirements for care and interventions.

### Provisions for post-trial care

When the trial is completed, the subjects will receive standardised treatment in accordance with guidelines.

### Outcomes

#### Primary outcomes

Wound healing time, wound exudation rate, cure rate during 14 days postoperation, recurrence rate during 3 months and total cure rate. The wound is regarded as cured when the grafted skin survives, and the wound has epithelised completely. Wound exudation will be rated on the basis of the number of times dirty underwear or sanitary pads are changed during 1 day.

#### Secondary outcomes

Operation duration, hospital stay length, postoperative scar scale and days to return to work. The duration of the operation is related to postoperative complications. Long hospital stays and days to return to work indicate that patients need to pay increased operation costs.

### Safety assessment

Before the study, the following safety indicators of the patients will be measured:

1. Laboratory tests, including routine blood tests (white blood cell count, total number of neutrophils, total number of lymphocytes, total number of monocytes, total number of eosinophils, total basophils, percentage of neutrophils, lymphocyte percentage, percentage of monocytes, eosinophil percentage, basophil percentage, total number of red cells, haemoglobin volume, erythrocyte specific volume, mean erythrocyte volume, mean erythrocyte haemoglobin content, mean erythrocyte haemoglobin concentration, erythrocyte distribution width, total platelet count, mean platelet volume, platelet distribution width and specific platelet volume); blood type (O Rh type); liver function tests (alanine transaminase and glutamyl transpeptidase); kidney function tests (serum creatinine); blood lipids; four blood coagulation indices (PT, APTT, TT and FIB); four infection diseases (antibodies of HIV, HBV, HCV and syphilis).
2. Imageological examinations, including chest X-ray and 12-lead electrocardiography. Enteroscopy will be performed to exclude surgical contraindications.
3. Vital signs, including blood pressure, respiration rate, heart rate, body temperature and physical examination.
4. The safety of the two operations will be assessed on the basis of postoperative wound pain, faecal continence and wound infection. Postoperative wound pain will be rated by using the Visual Analogue Scale. In this trial, the Wexner scale will be applied to estimate anal function. Postoperative wound infection will be described by using the wound infection risk score.
5. All adverse events occurring during the study, including mild, medium and severe impairments in patients, will be reported and recorded in detail by using a case report form (CRF).

### Participant timeline

The participant timeline is shown in Table 1.

**Table 1:** Schedule of enrolment, interventions and assessments

Time point		Enrolment	Allocation	Operation	Check-out
Enrolment	Eligibility screening	X			
	Informed consent	X			
	Allocation		X		
Intervention	Test group			X	
	Control group			X	
Assessment	Laboratory tests	X		X	X
	Vital signs	X		X	X
	Primary efficacies			X	
	Safety indicators			X	

### Sample size

This study is a superiority trial. Our pretest showed that the average wound healing times of the graft and no-graft groups were approximately 15 and 25 days, respectively. We assume that no difference in efficacy exists among the three centres. Assuming a two-sided significance level of 0. 05 and power of 80% (*α* = 0. 05, *β* = 0. 2), the sample size is calculated as 47 (fixed model as $n_{1} = n_{2} = \frac{{\sigma^{2} \left( {{\text{t}}_{\alpha } + t_{\beta } } \right)^{2} }}{{\delta^{2} }}$) [11]. Considering a loss to follow-up of 10%, the sample size for each group is 52 cases, and the total is 104 cases.

### Recruitment

This RCT, in which assessors and patients are blinded, will be conducted at three research centres. The subjects in this study will be recruited from outpatient and inpatient wards by placing advertisements on social media platforms and distributing posters with details of the study and contact information in public areas of hospitals.

Patients who meet the inclusion criteria will be invited to participate in the study and provided with the details of the RCT protocol. All patients will be required to sign an informed consent form and provide complete information. The patients will be randomised (1:1) to the test and control groups. None of the patients will know about their assigned group to keep the trial blind. Then, their baseline characteristics will be assessed, and a database record will be obtained by the same colorectal surgeon. All patients will be treated with surgery and followed up on the 3rd day, 7th day, 14th day, 21st day, 28th day, 2nd month, 3rd month and 6th month postoperation. After follow-up, pictures of the wounds will be taken, and data will be evaluated.

In addition, all of the patients will be given a schedule of intervention dates and follow-up appointments. These evaluations will be performed by an assistant who will be involved neither in the randomisation nor in the treatment.

## Assignment of interventions: allocation

### Sequence generation

A statistician who is independent and separate from the research team will use the SAS (SAS Institute Inc, Cary, NC, USA) randomisation programme to generate random numbers with simple randomisation.

### Concealment mechanism

Central randomisation will be used. The random and technique numbers will be unified. The randomisation programme will be created by a statistician from the School of Basic Medical Science of GZUCM. The randomly generated number will then be provided in a sealed envelope or online and can be accessed by the clinical trial manager. The participants will be assigned in accordance with the randomly generated number.

### Implementation

Random masking will be implemented through the double-blind design. When the patients are allocated, random numbers will be obtained through the central randomisation system. Researchers and patients will know only the random number to avoid the destruction of random grouping.

## Assignment of interventions: blinding

### Who will be blinded?

A total of 104 eligible subjects will be randomly assigned to two parallel groups. Statisticians will adopt the SAS randomisation programme to generate a randomisation list. Then, the clinical trial manager will divide the patients into two groups in accordance with the randomisation list. All people involved, including the investigators, will be blinded to the assignment of the subjects. The relevant procedures and allocations will be given to the patients by the surgeons in accordance with the randomly generated numbers at different stages. All the study field personnel, patient, sponsor and contract research organisation will remain blinded in the study.

### Procedure for unblinding if needed

Unblinding meetings will be held. Unblinding in emergency situations will also be implemented through the central randomisation system.

## Data collection and management

### Plans for the assessment and collection of outcomes

A CRF will be used to assess and collect outcomes and baseline information. In addition, the clinical trial database will be constructed by a designated data manager who will be responsible for regular database management and maintenance. All data will be imported into the clinical trial database by two research assistants. The investigator will be responsible for maintaining accurate, complete and up-to-date records for each subject. The investigator will also be responsible for maintaining any source documentation related to the study, including any films, tracings, computer discs or tapes.

The anonymity of the participating subjects will be maintained. For data collection and management purposes, the subjects will only be identified by subject numbers. Documents that identify the subject beyond the subject number will not be submitted to the sponsor (e.g. the signed informed consent document and subject initials) and will be maintained in strict confidence by the investigator except to the extent necessary to allow auditing by regulatory authorities, study monitor or sponsor representatives.

Field personnel will record all data for each study subject on electronic CRFs (eCRFs) by using an electronic data capture (EDC) system. A study procedure manual for additional information regarding CRFs will be used as source documentation. The eCRFs will be completed on-site in a timely manner, and the investigator will promptly review the completed eCRFs for each subject after every visit. As the person is ultimately responsible for the accuracy of all eCRF data, the investigator will sign the Investigator’s Statement in each subject’s eCRF.

### Plans to promote participant retention and complete follow-up

Follow-up will be conducted at baseline, at 1 week ± 3 days, 2 weeks ± 3 days, 3 weeks ± 3 days, 4 weeks ± 3 days, 8 weeks ± 3 days and 12 weeks ± 3 days and 24 weeks ± 3 days. Any missing or incorrect data will be detected by the software system. In such a case, the original CRFS will be checked to correct or complete every piece of data.

### Data management

The clinical trial database will be constructed by a designated data manager who will be responsible for regular database management and maintenance. All data will be imported into the clinical trial database by two research assistants. The EDC system automatically generates queries resulting from the computer checks embedded into the system to ensure the accuracy, quality, consistency and completeness of the database. Manual queries resulting from review by monitors, medical coders and other data management staff will also be generated from within the EDC system, where they are tracked. Queries can be resolved, and data entered can be corrected on-site when necessary. Every change to the data will be captured in the EDC system audit trail. Upon the completion of the study or after reaching a prespecified point in the study, data management will lock the database and generate the SAS datasets necessary for data analysis and reporting.

### Confidentiality

The confidentiality measures are as follows: The results of this research project may be published in medical journals. The patient’s information will be represented by a unique number, and the coded information will be stored at the First Hospital of GZUCM. The information of subjects will be maintained confidential as required by law. However, the records of subjects may be reviewed to ensure that the study complies with applicable laws and regulations.

### Plans for collection, laboratory evaluation and storage of biological specimens for genetic or molecular analysis in this trial/future use

Not applicable.

## Statistical methods

### Statistical methods for primary and secondary outcomes

SPSS 22. 0 (IBM, Armonk, NY, USA) software will be utilised for statistical analysis. *P* ≤ 0. 05 will be considered statistically significant.

The primary outcomes will be compared between groups by using Pearson’s chi-squared test. *t*-test, corrected *t*-test (equal variance not assumed), and analysis of variance for repeated measurements will be used for data analysis, and grade data will be assessed via Wilcoxon two-sample test.

### Interim analyses

Colorectal surgeons will have access to interim results and make the final decision to terminate the trial.

### Methods for additional analyses

The hybrid control will use multivariate logistic regression and odds ratio and 95% confidence interval estimation. Clinically significant variables from the univariate analysis will be included in the multivariate model. Goodness-of-fit will be evaluated through the Hosmer–Lemeshow test. Statistical analysis will be performed by using SPSS 22.0. A two-tailed significance level of 0.05 will be used for all tests. *P* < 0.05 will indicate statistical significance.

### Analysis methods for handling protocol nonadherence and any statistical methods for handling missing data

Study populations include the intent-to-treat analysis set defined as all randomised patients and the per-protocol analysis set defined as all patients in the intent-to-treat population without any major protocol deviations. Multiple imputations will be used to manage missing values.

### Plans to give access to the full protocol, participant-level data and statistical codes

Data, *the full protocol and statistical code* are available upon reasonable request. All inquiries about data sharing should be directed to drliyuying@126.com.

## Oversight and monitoring

### Composition of the coordinating centre and trial steering committee

The steering committee consists of three members, including two senior colorectal surgeons and a statistician, who will supervise the trial and ensure data safety and quality. The committee is independent of the research team and has no conflict of interest with the investigators. The committee will provide regular supervision, hold monthly meetings and organise a field trip at least once to ensure that the trial is performed smoothly and ethically. Moreover, a supervisor will ensure the authenticity and integrity of the data. During the visit, the steering committee will interview the investigators, check the original research documents and the registration of subjects and confirm whether the clinical centre complies with the research protocol. Any noncompliance with the agreement will be fully recorded by using a violation report form. Furthermore, they will identify the problems in the trial and propose suggestions for the modification of the protocol. If any decision to amend the protocol has to be made, the approval will be sent to the institutional medical ethics committee in writing, and the investigator will be notified in writing after approval. The protocol will be immediately updated in the system.

### Composition, role and reporting structure of the data monitoring committee

The data monitoring committee will include three members, namely an EDC system administrator, a data administrator and a statistician. The committee is independent of the research team and has no conflict of interest with the investigators. They will perform data management design, processing and regulation, quality control, security and confidentiality measures and EDC system management.

### Adverse event reporting and harm

#### Observation and recording

Any adverse reaction during the study period will be recorded on the adverse event form. Follow-up investigation will include detailed records of the treatment process and results and will be conducted until the laboratory examination returns to normal and the symptoms and signs disappear.

#### Medical treatment

When an adverse reaction is discovered, the investigator will decide on the diagnosis and treatment measures on the basis of the condition. They will also decide whether to terminate the observation. In the event of a serious adverse event, the unit undertaking the clinical research will immediately take necessary measures to protect the safety of the subjects.

#### Reporting of adverse events

The investigator will evaluate each adverse event, and if a serious adverse event occurs, it will be reported in accordance with the requirements of serious adverse event reporting.

All adverse events will be recorded on the adverse event page of the medical record report form. For each adverse event, the duration, severity and test drug, including the relevance and necessity to take measures as well as the taken treatments, will be described.

#### Requirements for reporting serious adverse events

If a serious adverse event occurs, the investigator will take appropriate treatment/rescue measures for the subject in accordance with his own clinical judgement to protect the safety of the subject.

The investigator should notify the main investigator and the ethics committee of the clinical research institution by the fastest means of communication within 24 h after learning of the serious adverse event. The investigator will submit the completed serious adverse event report form to the lead unit and the main investigator, who will file it with the team leader’s ethics committee within 3 days of being informed.

#### Frequency and plans for trial auditing

The trial conduct will be audited every 2 months.

#### Plans for communicating important protocol amendments to relevant parties (e.g. trial participants and ethical committees)

Committees will identify the problems in the trial and provide suggestions for the modification of the protocol. If any decision to amend the protocol is to be made, a written application will be submitted to the institutional ethics committee, and the investigator will be notified in writing after approval. The protocol will be immediately updated in the system.

#### Dissemination plans

All presentations will protect the integrity of the primary research objectives. None of the data that compromise blinding will be released before results are available. The steering committee will discuss the recommendations on the timing of these final data that may be presented at the meetings. The primary outcomes will be published in abstract books and articles.

## Discussion

Anal fistulas are common problems in the clinic. Although they have been investigated intensively by many surgeons, studies on anal fistulas remain insufficient. Some highly complex fistulas remain surgically challenging. Long postoperative wound healing times continue to be a problem for anal fistulas. Numerous techniques have been developed for anal fistula treatment. This situation shows that while colorectal surgeons have made great efforts to find a superior method for treating anal fistulas, no “gold standard” methods for this common clinical problem exist. Surgical options mainly vary depending on the preoperative classification of fistulas [12]. Eliminating all sepsis completely and permanently, achieving healing and minimising the incidence of recurrence while preserving anal function and continence are the aims of anal fistula surgery [13]. Shortening wound healing time is another important aim.

Fistulas are laid open to allow healing to start from the base [9]. Skin grafting has been shown to be an effective treatment for the rapid coverage of ulcers. Therefore, we will apply skin grafting for the wound healing of simple and low anal fistulas. Because for anal fistulotomy, the simpler the fistula, the less muscle is cut, and the more proximally the tract crosses the sphincter, the higher risk of impairing the continence. Low simple intersphincter anal fistulas are the ideal indication for skin harvesting and grafting at the surgical wound in one stage. Harvesting skin at the surgical wound avoids impairing other parts of the body and is, therefore, minimally invasive for patients. The indicators that we chose for this trial are widely used to describe and assess the efficacy and safety of anal fistula treatment. We anticipate that our results will show that the one-stage SSG of surgical wounds is effective for treating low simple intersphincter anal fistulas. Our supposition will be confirmed by this study.

## Trial status

The recruitment began on 25 March 2021, and we expect that this study will be completed by 24 October 2022.

## Data Availability

The data used to support the findings of this study will be available from the Chinese Clinical Trial Register (ChiCTR2000039174) within 6 months after the trial is completed.

## Abbreviations

SSG: Shaped skin grafting
RCT: Randomised controlled trial
GZUCM: Guangzhou University of Chinese Medicine
MRI: magnetic resonance imaging
CRF: Case report form
eCRF: Electronic case report form
EDC: Electronic data capture
